# Mouse models for studying anti‐amyloid antibody‐induced ARIA

**DOI:** 10.1002/alz70861_108865

**Published:** 2025-12-23

**Authors:** Cynthia A Lemere

**Affiliations:** ^1^ Ann Romney Center for Neurologic Diseases, Brigham and Women's Hospital, Harvard Medical School, Boston, MA, USA; Brigham and Women's Hospital; Harvard Medical School, Boston, MA USA

## Abstract

**Background:**

Anti‐amyloid immunotherapy treatments for early‐stage Alzheimer’s disease (AD) have received FDA approval and are currently in use in the clinic. Vascular sides effects known as Amyloid‐Related Imaging Abnormalities (ARIA) are detected by MRI monitoring and include ARIA‐E (brain edema) and ARIA‐H (microhemorrhages and superficial siderosis). Risk factors for ARIA include carriage of ApoE4 alleles, pre‐existing vascular amyloid (cerebral amyloid angiopathy, CAA), hypertension, pre‐existing microhemorrhages, and anti‐thrombolytic use. Most ARIA‐E occurs early in treatment and is asymptomic however, while rare, severe cases of ARIA have occurred and in some cases were fatal. Because these events occur during treatment and typically are not fatal, it is difficult to discern the underlying pathophysiological changes occurring during and after the resolution of ARIA. Therefore, mouse models of amyloid deposition are used to study the mechanisms of ARIA.

**Method:**

This talk will provide an overview of the mouse models that have been shown to develop ARIA with anti‐amyloid antibody treatment to date and will highlight the advantages as well as the challenges and limitations of these animal models, including the requirement to age mice for 1‐2 years to develop CAA before starting treatment. Newer mouse models that develop CAA faster will be discussed that may help facilitate future ARIA studies in mice.

**Result:**

Thus far, studies of ARIA in amyloid mouse models have focused mainly on the development of microhemorrhages (ARIA‐H) following chronic immunization. However, MRIs and measures of BBB leakiness and RBC extravasation are now used to monitor the very early effects of anti‐amyloid immunotherapy in mouse brain. Recruitment of immune cells to the brain vasculature, activation of the classical complement cascade, and attempts at vascular remodeling in response to anti‐amyloid antibodies have been demonstrated in mice. Various ‐omics studies suggest potential biomarkers.

**Conclusion:**

Take together, studies of anti‐amyloid antibody‐induced ARIA in amyloid mouse models have provided insight into the mechanisms underlying ARIA. These models can be used to both identify therapeutic targets and test drugs for efficacy in mitigating ARIA that may be translated into human clinical studies. Funding: NIH NIA 1R01AG084531, NINDS 1R01NS136122‐01 and Cure Alzheimer Fund (CAL).

